# Supplementary material for: Differential Expression of Genes between a Tolerant and a Susceptible Maize Line in Response to a Sugarcane Mosaic Virus Infection
Source: Viruses. 2022 Aug 17;14(8):1803. doi: 10.3390/v14081803 (PMC9415032; doi:10.3390/v14081803)
Supplement: Supplementary file 1 [file viruses-14-01803-s001.zip › viruses-1849363-supplementary.pdf]

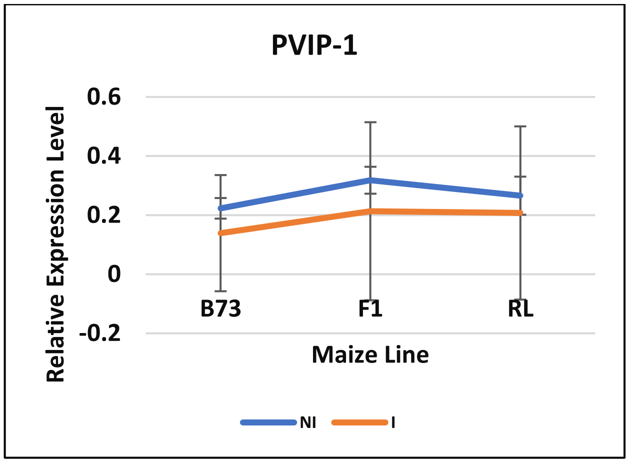
PVIP-1
Relative Expression Level
0.6
0.4
0.2
0
-0.2
B73
F1
RL
Maize Line
NI
I

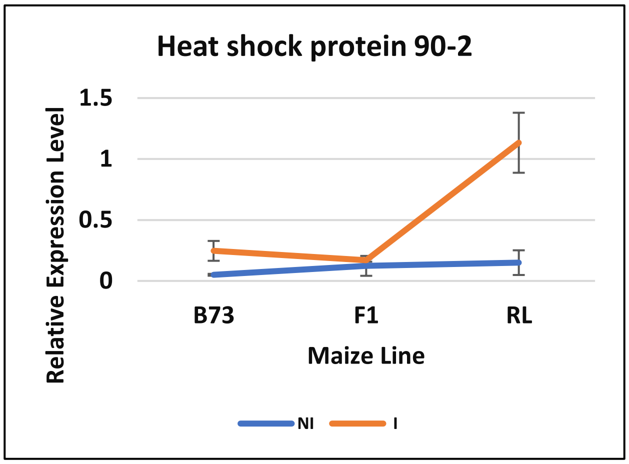
Heat shock protein 90-2
Relative Expression Level
1.5
1
0.5
0
B73
F1
RL
Maize Line
NI
I

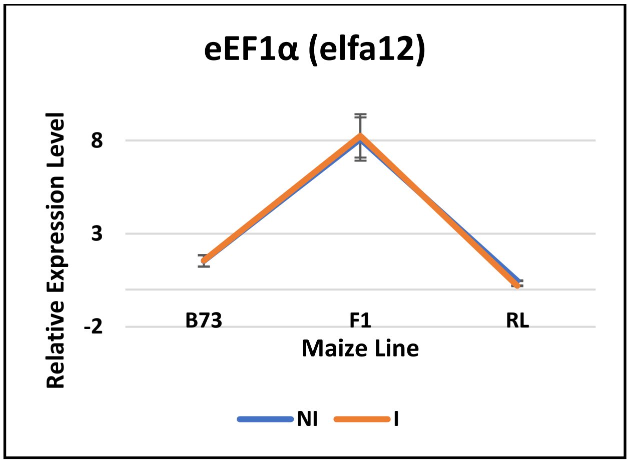
eEF1α (elfa12)
Relative Expression Level
8
3
-2
B73
F1
RL
Maize Line
NI
I

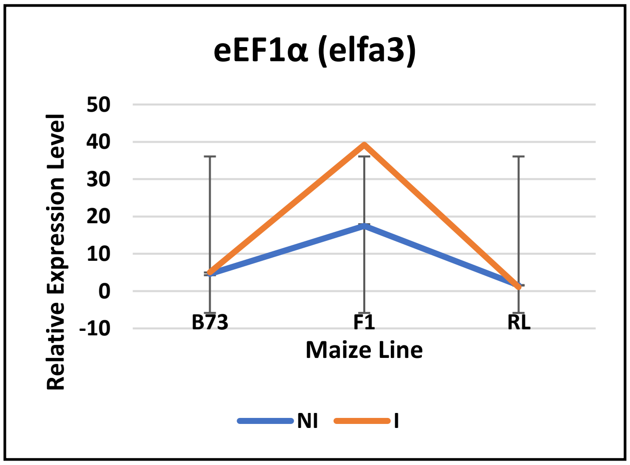
eEF1α (elfa3)
Relative Expression Level
50
40
30
20
10
0
-10
B73
F1
RL
Maize Line
NI
I

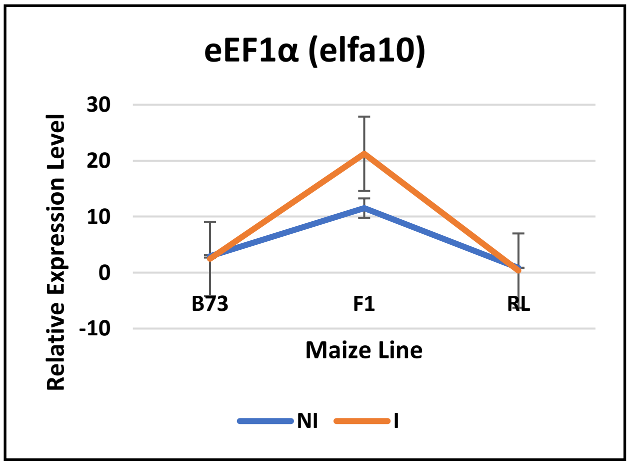
eEF1α (elfa10)
Relative Expression Level
30
20
10
0
-10
B73
F1
RL
Maize Line
NI
I

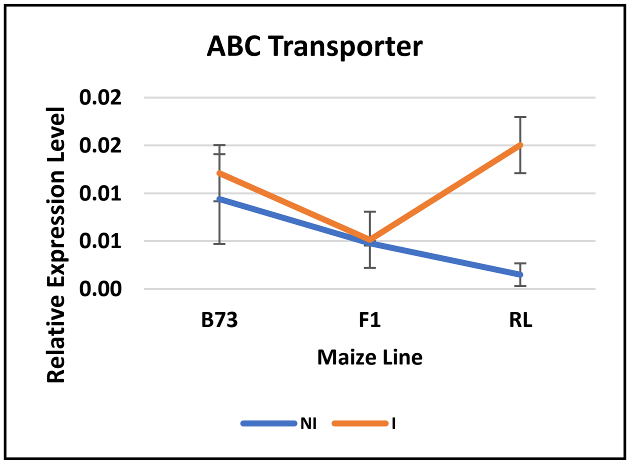
ABC Transporter
Relative Expression Level
0.02
0.02
0.01
0.01
0.00
B73
F1
RL
Maize Line
NI
I

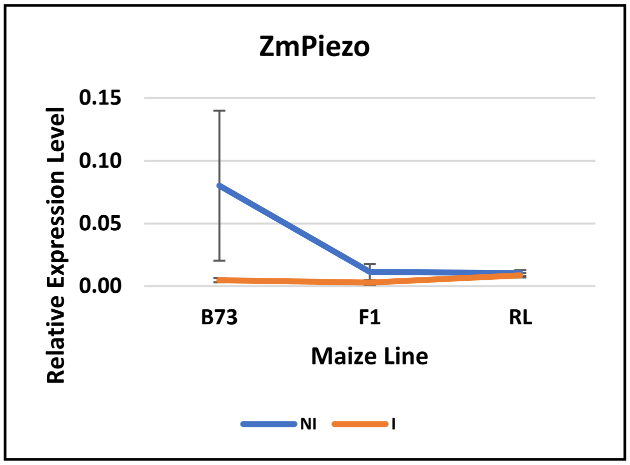
ZmPiezo
Relative Expression Level
0.15
0.10
0.05
0.00
B73
F1
RL
Maize Line
NI
I

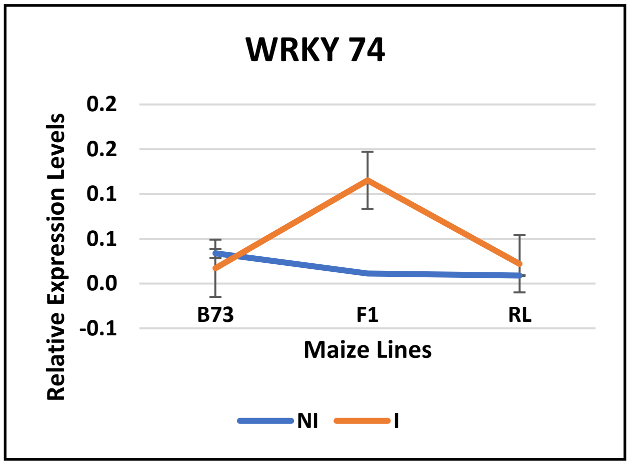
WRKY 74
Relative Expression Levels
0.2
0.2
0.1
0.1
0.0
-0.1
B73
F1
RL
Maize Lines
NI
I

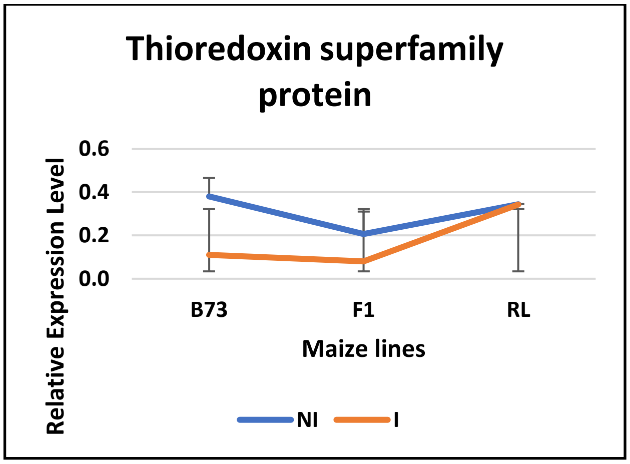
Thioredoxin superfamily protein
Relative Expression Level
0.6
0.4
0.2
0.0
B73
F1
RL
Maize lines
NI
I

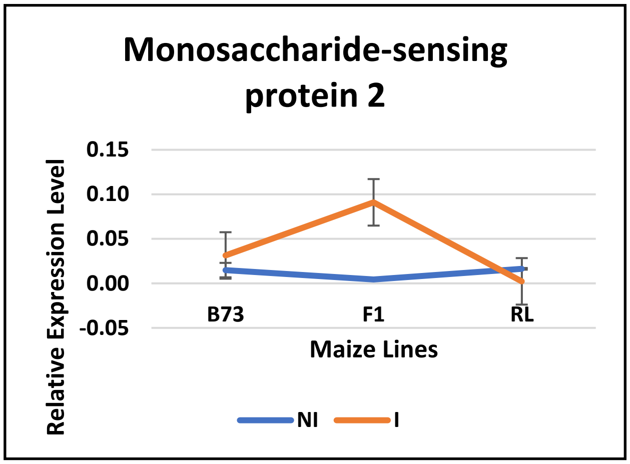
Monosaccharide-sensing protein 2
Relative Expression Level
0.15
0.10
0.05
0.00
-0.05
B73
F1
RL
Maize Lines
NI
I

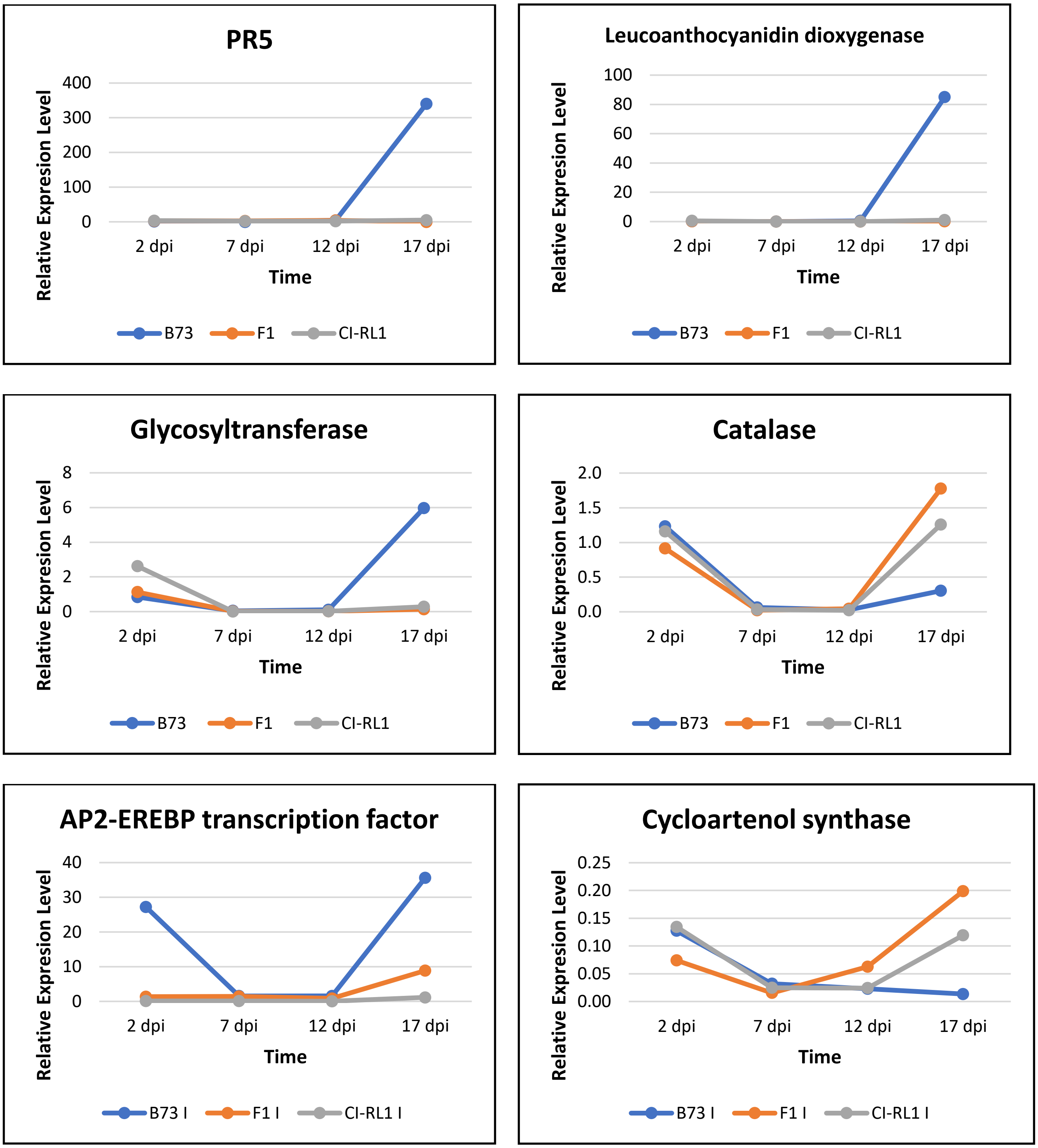

Figure S1. Relative expression levels of 10 genes obtained from Biological Process (BPs) and 6 from the comparison of different criteria. NI= non-SCMV inoculated/mock (blue); I= SCMV Inoculated (red).

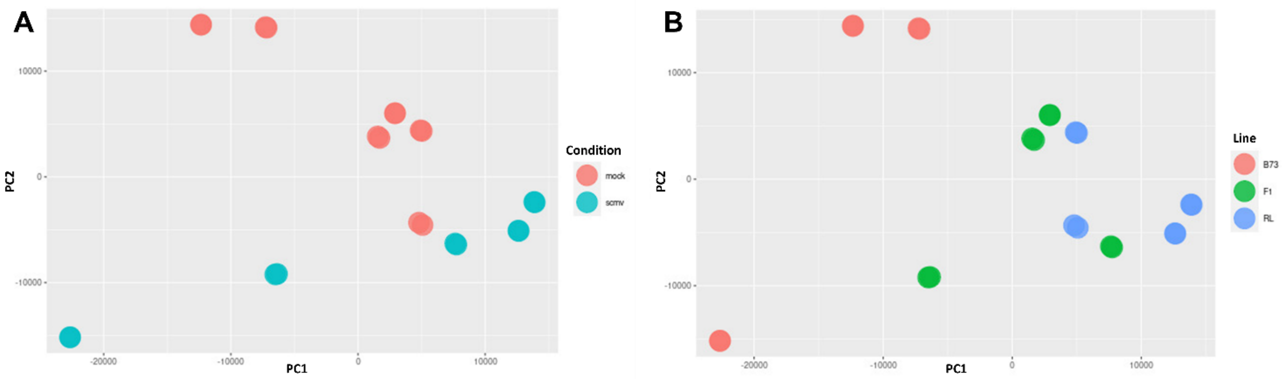

Figure S2. Principal components analysis (PCAs) of the libraries generated in this work. Libraries were analyzed by condition (**A**; mock in red or SCMV-inoculated in blue) or by line (**B**; B73 in red, F1 in green and CI-RL1 in blue)

Table S1. Eukaryotic initiation factors (eIFs) and eukaryotic elongations factors (eEFs) annotated in the MaizeGDB database. The corresponding gene model in B73 RefGen_v3 is cited under the "MaizeGDB GM" column. The gene models within the genomes Zm-B73-REFERENCE-GRAMENE-4.0 and Zm-B73-REFERENCE-NAM-5.0 are cited in the "Associated GMs" column. The "Arabidopsis Best hit" colum shows the information found in the "Annotations" section of the gene model in MaizeGDB.

Annotation	MaizeGDB GM	Associated GMs	Arabidopsis Best Hit
eukaryotic initiation factor 3 (eif-3)	GRMZM2G093050	Zm00001d039518<br>Zm00001eb121480	-
eucaryotic initiation factor4 (eif-4)	GRMZM2G116034	Zm00001d014673<br>Zm00001eb226920	-
eukaryotic initiation factor4a (eif-4a)	GRMZM2G027995	Zm00001d036480<br>Zm00001eb272920	-
eucaryotic translation initiation factor5 (eif-5)	GRMZM2G165917	Zm00001d000300<br>Zm00001eb208030	AT1G36730 (Translation initiation factor IF2/IF5)
eucaryotic initiation factor6 (eif-6)	GRMZM2G002616	Zm00001d041682<br>Zm00001eb137550	AT4G18040 (eIF4E)
eucaryotic initiation factor7 (eif-7)	GRMZM2G022019	Zm00001d014065<br>Zm00001eb222060	AT5G35620 (eIF(iso)4E)
eIF4E* (Not annotated)	GRMZM2G113096	Zm00001d041973<br>Zm00001eb139930	AT4G18040 (eIF4E)
elongation initiation factor 2 (eif-2)	GRMZM2G107654	Zm00001d021815<br>Zm00001eb324510	AT1G04170 (EIF2 GAMMA)
elongation initiation factor5A (eif-5a)	GRMZM2G113696	Zm00001d006760<br>Zm00001eb108410	AT1G13950 (EIF-5A)
Elfa1 - elongation factor α 1	GRMZM2G151193	Zm00001d009868<br>Zm00001eb346210	AT1G07930 (GTP binding Elongation factor Tu family protein)
elfa2 - elongation factor α 2	GRMZM2G149768	Zm00001eb285220	AT1G07940 (GTP binding Elongation factor Tu family protein)
elfa3 - elongation factor α 3	GRMZM2G154218	Zm00001d037873<br>Zm00001eb285210	AT1G07920 (GTP binding Elongation factor Tu family protein)
elfa6 - elongation factor α 6	GRMZM2G057535	Zm00001d021788<br>Zm00001eb324270<br>Zm00001eb324280	AT1G07930
elfa7 - elongation factor α 7	GRMZM2G110509	Zm00001d009870<br>Zm00001eb346230	AT1G07920
elfa9 - elongation factor 1α 9	GRMZM2G153541	Zm00001d046449<br>Zm00001eb385900	AT1G07930, AT1G07940
elfa10 - elongation factor 1α 10	GRMZM2G343543	Zm00001d036904<br>Zm00001eb276860	AT1G07920, AT5G60390
elfa11 - elongation factor α 11	AC233866.1_FG006	Zm00001d037905<br>Zm00001eb285450	AT1G07940
elfa12 - elongation factor 1-α-like 12	GRMZM2G001327	Zm00001d037877<br>Zm00001eb285230	AT1G07930, AT1G07940

Table S2. List of the resistance candidate genes searched for in the expression pattern clusters. The name of the gene (and its abbreviation), the organism, and the National Center for Biotechnology Information (NCBI) accesion number of the sequence that was used are also shown. Additionally, the alteration of the viral cycle related to the gene mutation is listed in the column "Resistance at". Finally, the corresponding candidate gene model in maize is provided in the "Best hit maizeGDB" column.

Gene		Organism	Resistance at	Sequence (GenBank)	Best hit maizeGDB
Synaptotagmin	*SYTA*	Arabidopsis	Cell-to-cell movement	NM_127668.4	GRMZM2G050193
Fibrillarin	*Fibrillarin*	Benthamian	Long-distance movement	AM269909	GRMZM2G150648
DEAD-box protein/Ded1p	*DED1*	Yeast	RNA replication	NC_001147.6	GRMZM2G403636
Re initiation supporting protein	*RISP*	Arabidopsis	Transactivation/viral amplification	NM_125513.1	GRMZM2G146416
Acyl coenzyme A binding protein 1	*ACBP1*	Arabidopsis	Activity of RNA replication complexes	AT5G53470.1	GRMZM2G108138
SNARE-Protein Syp71	*SYP71*	Arabidopsis	Mediating the fusion of viral vesicles with chloroplast	NM_111809	GRMZM2G057251
Chloroplast phosphoglycerate kinase	*Chl-PGK*	Benthamiana	Targeting of chloroplast	HQ450764.1	GRMZM2G083016
ADP ribosylation factor 1	*ARF1*	Arabidopsis	Virus replication	NM_102198.5	GRMZM2G395844
Sm motif protein, Lsm1p	*LSM1*	Yeast	Virus replication	NM_001181557.1	GRMZM2G021464
Glyceraldehyde-3-phosphate dehydrogenase	*GPDH*	Benthamiana	Virus replication	-	GRMZM2G046804
Heat shock protein 70	*Hsp70*	Benthamiana	Assemble of replicase complex/Enhance viral RNA replication	KX912913.1	GRMZM2G340251
Heat shock protein 90	*Hsp90*	Benthamiana	Assemble of replicase complex	LC314272.1	GRMZM2G012631
Prunus persica DEAD-box RNA helicase-like	*PpDDXL (eIF4E)*	Peach	Viral genome translation and replication	GQ865547.1	GRMZM2G027995
Poly (A)-binding protein 2	*PBAP2*	Arabidopsis	Virus accumulation	NM_119572	GRMZM2G352129
Poly (A)-binding protein 4	*PABP4*	Arabidopsis	Virus accumulation	NM_127899	GRMZM2G102829
Poly (A)-binding protein 8	*PBAP8*	Arabidopsis	Virus accumulation	NM_103863	GRMZM2G013619
Tobamovirus multiplication 1	*TOM1*	Arabidopsis	Efficient viral multiplication	NM_118299	GRMZM2G020281
Tobamovirus multiplication 2A	*TOM2A*	Arabidopsis	Efficient viral multiplication	NM_102974	GRMZM2G049422
Host Small GTP-binding Protein	*ARL8*	Arabidopsis	Viral RNA replication	NM_114847	GRMZM2G081622
Plasmodesmata-located protein	*PDLP*	Arabidopsis	Cell-to-cell trafficking	NM_123765.3	GRMZM2G066860
Pectin methyl esterase	*PME*	Tomato	Cell-to-cell movement	U49330.1	GRMZM2G136106
Myosin X-2	*Myosin*	Benthamiana	Intercellular movement	DQ875135.1	GRMZM2G435294
Actin	*Actin*	Benthamiana	Intercellular movement	JQ256516.1	GRMZM2G104017
Plasma-membrane associated cation-binding protein 1	*Pcap1*	Arabidopsis	Facilitates movement	NM_118145.4	GRMZM2G071089
COPII coatomer Sec24a	*Sec24a(ERMO2)*	Arabidopsis	Cell-to-cell movement	NM_111590.4	GRMZM2G081745
Chloroplast phosphoglycerate kinase	*cPGK2*	Arabidopsis	Virus accumulation	NM_104498	GRMZM2G047028
ROOT HAIR DEFECTIVE 3	*RHD3*	Arabidopsis	Cell-to-cell spread	NM_112241.4	GRMZM2G390374
TORTIFOLIA1/SPIRAL2	*TOR1*	Arabidopsis	Virus spread	NM_118840	GRMZM2G026309

Gene		Organism	Resistance at	Sequence (GenBank)	Best hit maizeGDB
TORTIFOLIA 2	*TOR2*	Arabidopsis	Virus spread	NM_100360	GRMZM2G152466
DNA-binding protein phosphatase 1	*DBP1*	Arabidopsis	Interaction with eIF4E/Virus accumulation	NM_128120	GRMZM2G158734
Cucumis melo Vacuolar Protein Sorting 41	*CmVPS41*	Melon	Viral loading in the phloem	MELO3C004827	GRMZM2G040247
Ethylene-inducible transcription factor RAV2	*RAV2*	Arabidopsis	Suppression of RNA silencing	NM_10555858	GRMZM2G169654
Calmodulin-related protein	*rgs-CaM*	Tobacco	Suppression of PTGS	AF329729.1	GRMZM2G104523
S-adenosyl-L-methionine synthetase	*OsSAMS1*	Rice	Enhanced production of ethylene	Os05g0135700	GRMZM2G117198
Rab GTPase	*AtRAN-F2b*	Arabidopsis	Colocalization of MP in pre-vacuolar late endosomal compartments	NM_118084	GRMZM2G131254
Protein Kinase	*CK2*	Tobacco	Viral replication/CP accumulation	AF374474.1	GRMZM2G047855
Catalase 1	*CAT1*	Benthamiana	Enhance virus accumulation	EU998969.1	GRMZM2G088212
Rice dwarf virus multiplication 1	*RIM1*	Rice	Virus multiplication/CP accumulation	Os03g0119966	GRMZM2G174070
Formate dehydrogenase	*FDH1*	Pepper	Reduced virus accumulation/systemic spread	CA02g29530	GRMZM2G049811
Calreticulin	*CTR3*	Arabidopsis	Reduced virus accumulation/Not infection observed in inoculated leaves	AT1G08450	GRMZM2G028516
Protein disulfide isomerase-like 5-1	*PDIL5*	Rice	Virus replication	XP_015628450.1	GRMZM2G073628
Movement Protein I 7	*MPI7*	Arabidopsis	Virus infectivity	N65789.1	GRMZM5G831519
Substrate bZIP60	*bZIP60*	Benthamiana	Viral pathogenesis	NM_001324734.1	GRMZM2G025812
Inositol-requiring protein-1 A	*IRE1A*	Arabidopsis	Viral symptoms delayed/Viral accumulation	NM_127306.4	GRMZM2G162167
Inositol-requiring protein-1 B	*IRE1B*	Arabidopsis	Viral symptoms delayed/Viral accumulation	NM_001203453.2	GRMZM2G137944
Essential for poteXvirus Accumulation 1	*EXA1*	Arabidopsis	Viral accumulation	NM_123660.3	GRMZM2G480002
Glucan synthase-like 4	*GSL4*	Arabidopsis	Plasmodesmata permeability	ACS36250.1	GRMZM2G326643
β-1,3-glucanase	*BG3*	Tobacco	Callose degradation	CAA82271.1	GRMZM2G335111
Tomato spotted wilt virus resistance 1	*TSW*	Pepper	Viral genome translation and replication	KT751527	GRMZM2G173647
eIF4E	*Sbm1*	Pea	Viral genome translation and replication	AY611423	GRMZM2G002616
eIF4E	*mo1/pot-1*	Lettuce	Viral genome translation and replication	AF530162	GRMZM2G002616
eIF4E	*L3*	Pepper	Viral genome translation and replication	AB523370	GRMZM2G038388
eIF4E	*pvr1*	Pepper	Viral genome translation and replication	ABV54210	GRMZM2G002616
eIF4E	*PVR4*	Pepper	Viral genome translation and replication	KT359375	GRMZM2G173647
Potyvirus VPg Interacting Protein	*PVIP1*	Pea	Systemic spread	NM_111657.4	GRMZM2G162786
Atypical thioredoxin h	*ZmTrx h*	Maize	Long distance movement	-	GRMZM2G014055
Piezo-like ion mechano-channel	*ZmPiezo*	Arabidopsis	Systemic movement	At2g48060	GRMZM2G329944

Table S3. Candidate genes found in group 1 (blue) and group 2 (red) of the expression pattern clusters. The viral cycle process affected is listed in column "Resistance at". A total of nine genes were found to interfere with viral translation (TT), four with cell-to-cell (CC) movement, and two with long-distance movement (LD).

Cluster	Gen	Resistant at	TT	CC	LD
C0 C1 C2	*BG3*	Callose degradation/ Cell-to-cell movement		1	
C0 C1	*EXA1*	Viral accumulation	1		
C0 C2	*LSM1*	Virus replication	1		
	PVIP	Systemic spread			1
C0	*PpDDXL*	Viral genome translation and replication	1		
	IRE1A	Viral symptoms delayed/Viral accumulation	1		
	ZmPiezo	Systemic movement			1
	RIM1	Virus multiplication/CP accumulation	1		
	Chl-PGK	Targeting of chloroplast	1		
C1	*RISP*	Transactivation/viral amplification	1		
	OsSAMS1	Enhanced production of ethylene			
C2	*Pcap1*	Facilitates movement		1	
	Myosin	Intercellular movement		1	
C3	*PBAP8*	Virus accumulation	1		
C4	*IRE1B*	Viral symptoms delayed/Viral accumulation	1		
	PDLP	Cell-to-cell trafficking		1	
		**Total**	9	4	2

Table S4. List of primers used in this work.

Primer name	Target	Sequence	
		Forward	Reverse
qABC	ABC transporter B family member 21	CCTCACTGGTTCTCTGGCTC	GAACGTCCTCCAGCTTCCTC
qBTub	Beta tubulin	CATCTGCTATGTTCCGTGGGA	CGAGTTGCCGATGAATGTGG
qE1A1	Elongation factor 1-alpha (*elfa12*)	CATTGATGCCCCTGGACAC	CAAGGAGAGCATGTTCACGG
qE1A2	Elongation factor 1-alpha (*elfa3*)	GCCAGCTTCACCTCCCAG	CACCATACCAGCATCACCG
qE1A3	Elongation factor 1-alpha (*elfa10*)	GAAGCGTGGGTATGTGGC	GCCAGACCGCCTATCAATC
qHS	Heat shock protein 90-2	GTGAGACCCTCCAGCAGAAC	GCAGCTCCGCAATCTTGTTC
qPVIP1	Homolog to Potyvirus VPg-Interacting Protein	CAGGTTGGCTCGCCTGACAT	TCCGCCGCCTTACTCACAAC
qMSP	Monosaccharide-sensing protein 2	CGGACGATATCTTGGAGGAC	CACAGTGGGCTCATTTTGC
SCMV-CP	SCMV-Ver1 Coat Protein	AACCAGTGGCTCAGGAAC	TTGTTCATCTCCGTCCATCA
qSCMV-CP	SCMV-Ver1 Coat Protein (for real time PCR)	GCCAAGATACGGACTTCAGC	CATGTGGGCTTCCTTAGCTC
qTxS	Thioredoxin superfamily protein	GTGTTGGTCGCACGTTCTTC	GTGCCCTCGTCTTTCCTTG
qWRK74	Probable WRKY transcription factor 74	GAAGGTGTGCTGGGAAGGAG	CCTCCACGAGAACTCATCAGC
qZmPiezo	Homolog to PIEZO-like protein	GTGCCTTGTCCTTTGCATTC	CACTCACGGCAATGGACTTC
qPR5	Pathogen Related Protein5	GCAGCCAGGACTTCTACGAC	GGGCAGAAGGTGACTTGGTAG
qLeu-dx	Leucoanthocyanidin dioxygenase	CTCACTCGCCATGTTCTACG	CTCTGTCCTTCCGCAATTC
qGly	Glycosyltransferase	GTGGTACACGAGGAGGAAGC	GCCGCAGTGACTCATGAAC
qCat	Catalase	GGTTCGCCGTCAAGTTCTAC	CGAGAAGAAGTCGAGGATGC
qAP2	AP2-EREBP transcription factor	GGGACATGGACTTGGACTTG	GTTGGTGGAGCTTATTGCTG
qCAS	Cycloartenol synthase	GAGGGCGAGAAGACAACAAG	CTTCGGATTGCTGAGGAAG
